# Heme Induces IL-6 and Cardiac Hypertrophy Genes Transcripts in Sickle Cell Mice

**DOI:** 10.3389/fimmu.2020.01910

**Published:** 2020-08-21

**Authors:** Oluwabukola T. Gbotosho, Maria G. Kapetanaki, Samit Ghosh, Flordeliza S. Villanueva, Solomon F. Ofori-Acquah, Gregory J. Kato

**Affiliations:** ^1^Division of Hematology-Oncology, Department of Medicine, University of Pittsburgh School of Medicine, Pittsburgh, PA, United States; ^2^Department of Medicine, Pittsburgh Heart, Lung, Blood, and Vascular Medicine Institute, University of Pittsburgh School of Medicine, Pittsburgh, PA, United States; ^3^Division of Pulmonary, Allergy and Critical Care Medicine, Department of Medicine, University of Pittsburgh School of Medicine, Pittsburgh, PA, United States; ^4^Department of Medicine, Center for Translational and International Hematology, School of Medicine, University of Pittsburgh, Pittsburgh, PA, United States; ^5^Center for Ultrasound Molecular Imaging and Therapeutics, Heart and Vascular Institute, Pittsburgh Heart, Lung, Blood and Vascular Medicine Institute, University of Pittsburgh, Pittsburgh, PA, United States; ^6^School of Biomedical and Allied Health Sciences, University of Ghana, Accra, Ghana

**Keywords:** heme, hemolysis, sickle cell disease, IL-6, inflammation, cardiac hypertrophy genes

## Abstract

Emerging data indicate that free heme promotes inflammation in many different disease settings, including in sickle cell disease (SCD). Although free heme, proinflammatory cytokines, and cardiac hypertrophy are co-existing features of SCD, no mechanistic links between these features have been demonstrated. We now report significantly higher levels of IL-6 mRNA and protein in hearts of the Townes sickle cell disease (SS) mice (2.9-fold, *p* ≤ 0.05) than control mice expressing normal human hemoglobin (AA). We find that experimental administration of heme 50 μmoles/kg body weight induces IL-6 expression directly *in vivo* and induces gene expression markers of cardiac hypertrophy in SS mice. We administered heme intravenously and found that within three hours plasma IL-6 protein significantly increased in SS mice compared to AA mice (3248 ± 275 vs. 2384 ± 255 pg/ml, *p* ≤ 0.05). In the heart, heme induced a 15-fold increase in IL-6 transcript in SS mice heart compared to controls. Heme simultaneously induced other markers of cardiac stress and hypertrophy, including atrial natriuretic factor (Nppa; 14-fold, *p* ≤ 0.05) and beta myosin heavy chain (Myh7; 8-fold, *p* ≤ 0.05) in SS mice. Our experiments in Nrf2-deficient mice indicate that the cardiac IL-6 response to heme does not require Nrf2, the usual mediator of transcriptional response to heme for heme detoxification by heme oxygenase-1. These data are the first to show heme-induced IL-6 expression *in vivo*, suggesting that hemolysis may play a role in the elevated IL-6 and cardiac hypertrophy seen in patients and mice with SCD. Our results align with published evidence from rodents and humans without SCD that suggest a causal relationship between IL-6 and cardiac hypertrophy.

## Introduction

Sickle cell disease (SCD) is a complex hematological disorder that affects ~100,000 Americans and millions of people worldwide, especially in sub-Saharan Africa and India (1). Hemolysis and chronic inflammation are major components of the pathophysiology of SCD. Hemolysis is caused by erythrocyte injury due to secondary defects in erythrocyte fragility, deformability and increased endothelial adhesion resulting in release of hemoglobin and heme (2, 3). Chronic inflammation in SCD is partly due to leukocytosis with the abnormally high leukocytes and monocytes that secrete proinflammatory cytokines. In addition, products of hemolysis act as damage-associated molecular patterns (DAMPs) potentiating activation of many inflammatory mechanisms (4). Additionally, products of intravascular hemolysis such as free hemoglobin and arginase-1 impair nitric oxide bioavailability, endothelial function and organ function in SCD (2). Limited data indicate that heme can induce production of proinflammatory cytokines such as interleukin-6 (IL-6) by stimulating immune responses and inflammatory reactions (5). Hemolysis and inflammation are components of a wide spectrum of other clinical conditions including sepsis (6, 7), malaria (8, 9), and preeclampsia (10, 11). Common to patients with all these syndromes is an increased risk of cardiac dysfunction (12–15). Notable among proinflammatory cytokines elevated in SCD is IL-6. Serum levels of IL-6 are elevated both at steady state and during vaso occlusive crisis in both children and adults with SCD (16–20), concurrently with severe anemia and increased markers of hemolysis. Furthermore, IL-6 is associated with cardiomyopathies such as cardiac hypertrophy and fibrosis in experimental animals (21, 22) and in the general human population (23, 24). Importantly, cardiopulmonary complications are one of the leading causes of death in SCD (25, 26). This accounts for about 26% of deaths in adults with SCD (27), with left ventricular hypertrophy (LVH) found in over 60% of children and 37% in adults with SCD (28, 29). No prior publications have investigated any potential linkage between IL-6 and hemolysis in mice and patients with SCD, particularly in cardiac disease. In this study, we assess direct heme induction *in vivo* of IL-6 and genes relevant to cardiac hypertrophy in the heart of sickle cell mice. Our study shows that IL-6 is highly expressed in the circulation and in the heart of sickle cell mice at steady state. Furthermore, administration of extracellular heme further increased IL-6 and cardiac hypertrophy genes expression. To gain insight of the mechanism by which heme induces IL-6, we investigated the role of nuclear factor (erythroid derived 2)-like 2 (Nfe2L2 or Nrf2). Nrf2 is the master regulator of the cellular oxidative defense system and plays a significant role in the regulation of multiple heme-induced genes (30, 31).

## Materials and Methods

### Mouse Strains and Treatment

Male and female Townes' knocked-in transgenic sickle mouse (SS) and strain controls expressing normal human Hb (AA mice), C57BL/6J (*Nrf2*^+/+^) and *Nrf2*^−/−^ mice were used. C57BL/6 mice were obtained from the Jackson Laboratory (stock #000664) while SS, AA and *Nrf2*^−/−^ mice were obtained from a colony maintained by Dr. Solomon Ofori-Acquah's laboratory in our institution. Mouse genotypes were confirmed by PCR. Hemin [Fe(III)PPIX, Sigma-Aldrich, St. Louis, MO] was prepared as described elsewhere (32, 33). Freshly prepared hemin solution was protected from light and injected into 12–16 week old mice. A range of doses and times were tested and 3 h after injection produced consistent survival with no adverse effects on all strains of mice in this study. The mice were injected in the tail vein with a hemin dose of 50 μmoles/kg body weight for SS and AA mice, and 120 μmoles/kg body weight for *Nrf2*^+/+^ and *Nrf2*^−/−^ mice. Higher doses were needed for Nrf2^+/+^ and Nrf2^−/−^ mice to be able to neutralize the endogenous hemopexin and other heme-binding proteins and mimic the increase in circulating heme in chronic hemolysis. This allows comparable assessment of the transcriptional response. Control mice received sterile vehicle containing 0.25M NaOH adjusted to pH 7.5 with HCl used in preparation of hemin.

### Plasma Analysis

Freshly collected blood samples were centrifuged at 1,200 × g for 15 min to separate blood plasma. Plasma IL-6 concentration was measured using the mouse IL-6 ELISA kit (Sigma-Aldrich) following the manufacturer's instructions.

### Real-Time PCR

Whole organs were harvested from mice 3 h after hemin injection. Freshly isolated organs (300 mg) were snap-frozen and kept at −80°C until use. Organs were homogenized in Qiazol lysis reagent using the Next Advance Bullet Blender (Next Advance, Inc. Troy, NY). Clear lysates were obtained by centrifuging homogenized samples at 18,800 × g for 10 min. All tissue processing was carried out at 4°C. Total RNA was extracted from the tissue lysates using the miRNeasy Mini Kit (#217004, QIAGEN, Germantown, MD) and quantified using the Nanodrop 8000 microvolume spectrophotometer (ThermoFisher Scientific). Real-time PCR reactions were set-up in duplicates using 50 ng of RNA. Genes of interest were evaluated using the TaqMan® Gene expression assay (ThermoFisher) and the TaqMan® RNA-to-Ct™ 1-Step Kit (ThermoFisher) according to the manufacturer's instructions. Relative quantification was calculated with the standard ΔΔCt method; amplification signals from target gene transcripts were normalized to those from beta-glucuronidase (*Gusb*) transcripts. Relative fold induction was calculated by further normalization to gene transcripts from vehicle treated animals. *Gusb* gene expressions were similar across all mouse strains used and across all organs within a given mouse strain. *Gusb* gene expression in organs from control mice was similar to that from the corresponding organs from hemin-injected mice. We have previously published this in different organs from SS mice here (32) and in the heart of AA control mice in Supplementary Figure 1 in this study.

### IL-6 Protein Quantification

Mice were perfused with phosphate-buffered saline under anesthesia. Harvested organs (300 mg) were homogenized in RIPA buffer using the Next Advance Bullet Blender (Next Advance, Inc. Troy, NY). Homogenized samples were centrifuged at 18,800 x g for 10 min to obtain clear lysates. All tissue processing was carried out at 4°C. Heart IL-6 concentration was measured using the mouse IL-6 ELISA kit (Sigma-Aldrich) following the manufacturer's instructions. Total protein was quantified in the lysates using the BCA assay kit (ThermoFisher Scientific, #23225).

### Statistical Analysis

GraphPad Prism 7 software was used for all statistical analyses. Results are reported as mean ± SEM. Group means were compared using parametric tests, such as *t*-test (for 2 groups) and One-way ANOVA for more than two conditions. Statistical significance was set at *p* values of < 0.05.

## Results

### High Basal Expression and Heme-Induced Cardiac IL-6 in Sickle Cell Mice

**Figure 1:**
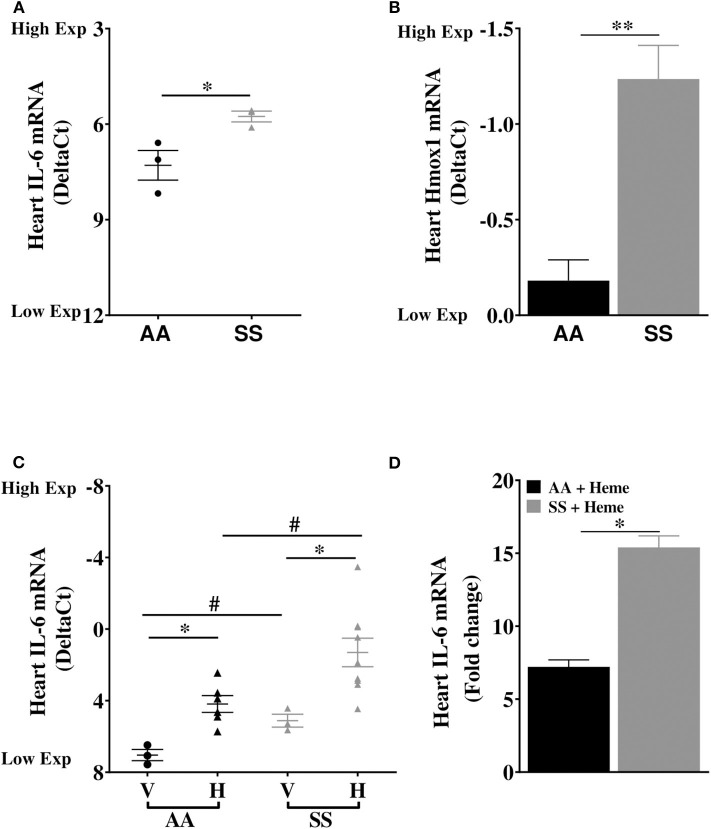
Cardiac expression of IL-6 and Hmox1 in SS mice. Heart mRNA expression of **(A)** IL-6 and **(B)** Hmox1 in naïve in 14 week old SS mice compared to age-matched AA mice (*n* = 3). The SS mice Hmox1 data was published here (32). **(C)** Heme induced cardiac IL-6 mRNA in SS and AA mice (*n* = 3–10). **(D)** Relative fold change in heme-induced cardiac IL-6 mRNA expression in heme treated AA and SS mice (*n* = 6–10). For DeltaC_t_, lowest value = highest expression and highest value = lowest expression. Target gene transcripts were normalized to Gusb for all mRNA expression levels. Gusb expression was similar in all mice strains used and in all of these organs in animals injected with either vehicle or hemin. For relative fold change, samples were further normalized to vehicle control gene transcripts. Unpaired Student's *t*-test or one-way ANOVA. Error bars indicate SEM. *p ≤ 0.05; **p ≤ 0.01. #*p* < 0.05 AA vs. SS. V, Vehicle and H, Heme.

**Figure 2:**
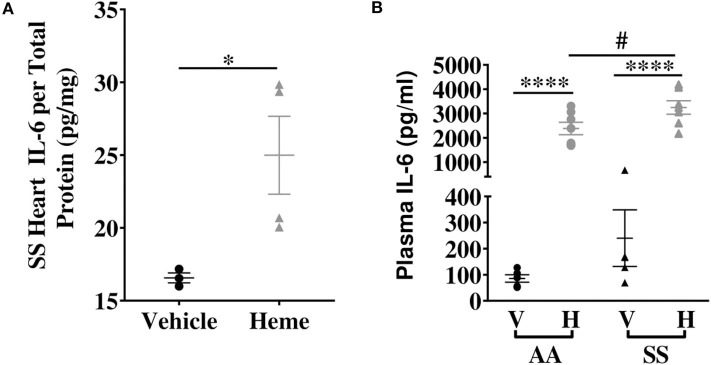
Heme induces IL-6 protein with a more pronounced effect in SS mice compared to AA controls. **(A)** Heme induced cardiac IL-6 protein in SS mice (*n* = 3–4). **(B)** Plasma IL-6 in SS and AA mice injected (IV) with 50 μmoles/kg body weight heme or vehicle (*n* = 5–7). Unpaired Student's *t*-test or one-way ANOVA. Error bars indicate SEM. *p < 0.05, ****p < 0.0001. #*p* < 0.05 AA vs. SS. V, Vehicle and H, Heme.

We investigated the basal expression of IL-6 transcripts in the heart of Townes SS mice and AA control mice. IL-6 expression was 2.9-fold higher in the heart of Townes SS mice compared to AA controls (Figure 1A, *p* ≤ 0.05). Hmox1 expression was significantly elevated in the heart (2.1-fold) of untreated Townes SS mice compared to AA controls (Figure 1B, *p* ≤ 0.01). We tested the hypothesis that products of hemolysis, specifically heme, would promote IL-6 expression in the AA heart to mimic the SS steady state expression. Injection of heme as previously described (32, 34) increased cardiac IL-6 transcript expression to levels comparable to vehicle-treated SS mice (Figure 1C, *p* < 0.05). This suggests that heme release in SS mice may be the critical factor that stimulates high SS basal IL-6 expression. SS mice were even more responsive to heme injection, with cardiac IL-6 transcripts rising 15.4-fold higher in heme-treated SS mice compared to vehicle controls (Figure 1D, *p* ≤ 0.05) and by about 53% in SS mice compared to AA mice controls (Figure 1C, *p* ≤ 0.001). We confirmed these mRNA results with analysis of IL-6 protein, which documented a 34% increase in IL-6 protein in the heart of SS mice injected with heme compared to vehicle controls (Figure 2A, *p* ≤ 0.05). These data support a role of heme in cardiac IL-6 regulation at steady-state and during acute heme increase in sickle cell disease.

### Increased Levels of Circulating IL-6 in Heme-Treated Sickle Mice

Elevated heme (3, 35) and IL-6 (16, 18) have been individually reported in the serum of SCD patients. We hypothesized that increase in extracellular heme rapidly upregulates IL-6 in the plasma of SS and AA mice. Heme treatment significantly increased plasma IL-6 protein levels about 25-fold in both AA and SS mice 3 h after heme injections. The heme-induced IL-6 level was significantly higher in SS mice than AA mice (3249 ± 276 vs. 2385 ± 256 pg/ml, *p* ≤ 0.05, Figure 2B). These data indicate a role for free heme in systemic regulation of IL-6.

### Heme-Induced Cardiac IL-6 Expression Is Negatively Regulated via Nrf2 Pathway

**Figure 3:**
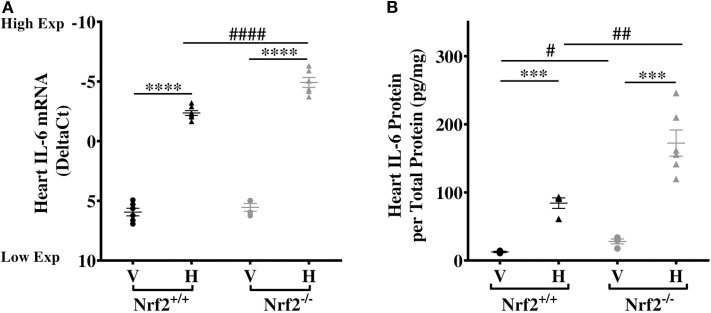
Nrf2 is not required for heme-induced cardiac IL-6. Heme-induced cardiac IL-6 in Nrf2^+/+^ and Nrf2^−/−^ mice. **(A)** RNA **(B)** Protein. Nrf2^+/+^ and Nrf2^−/−^ mice were injected with vehicle or heme (120 μmoles/Kg body weight). For DeltaC_t_, lowest value = highest expression and highest value = lowest expression. Target gene transcripts were normalized to Gusb for all mRNA expression level. Gusb expression was similar in all mice strains used and in all of these organs in animals injected with either vehicle or hemin. This dose was selected after standardization for producing consistent survival with no adverse effects on both strains of mice. One-way ANOVA. Error bars are SEM. ***p ≤ 0.001; ****p ≤ 0.0001, vehicle vs. heme within strain. #*p* ≤ 0.05 ##*p* ≤ 0.01 Nrf2^+/+^ vs. Nrf2^−/−^ (*n* = 3–7; 14–16 weeks old). V, Vehicle and H, Heme.

Our recent studies confirmed that the Nrf2 pathway mediates heme induction of cardiac Hmox-1 expression in SS mice (32), human monocytes (36), and keratinocytes (37). This led us to investigate whether the same pathway regulates cardiac IL-6 expression and its response to heme. We find no significant differences in cardiac IL-6 mRNA expression in vehicle-treated Nrf2^+/+^ and Nrf2^−/−^ mice (Figure 3A). Treatment with heme significantly augmented cardiac IL-6 mRNA levels in both strains (Figure 3A, *p* ≤ 0.001). Unexpectedly, IL-6 mRNA rose significantly higher in the hearts of the heme-treated Nrf2-deficient mice compared to the heme-treated Nrf2^+/+^ control mice. Confirming this mRNA finding, cardiac IL-6 protein was about 51% higher in heme-treated Nrf2^−/−^ mice compared to heme-treated Nrf2^+/+^ mice (*p* < 0.01, Figure 3B). The result shows that Nrf2 is not required for heme induction of cardiac IL-6 expression.

### Heme Upregulates Markers of Cardiac Hypertrophy in Sickle Mice

**Figure 4:**
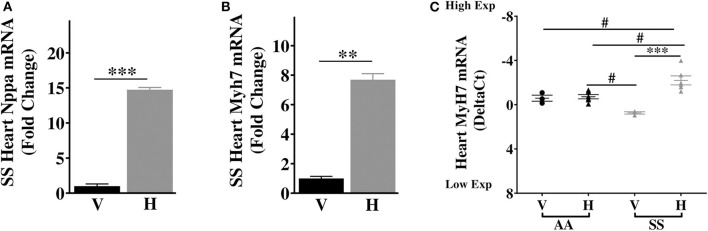
Heme induces high expression of transcripts of cardiac hypertrophy markers in SS mice. Heme induced the expression of **(A)** Nppa **(B)** Myh7 in the heart of SS mice 3 h after heme or vehicle injection **(C)** Myh7 in the heart of SS and AA mice 3 h after heme (50 μmoles/kg body weight) or vehicle injection. For DeltaC_t_, lowest value = highest expression and highest value = lowest expression. Target gene transcripts were normalized to Gusb for all mRNA expression level. Gusb expression was similar in all mice strains used and in all of these organs in animals injected with either vehicle or hemin. For relative fold change, samples were further normalized to vehicle control gene transcripts. Unpaired student's *t*-test and one-way ANOVA. Error bars indicate SEM. ***p ≤ 0.001; **p ≤ 0.001, vehicle vs. heme within strain. #*p* ≤ 0.05 AA vs. SS (*n* = 3–8; 14–16 weeks old). V, Vehicle and H, Heme.

Our results above showed elevated basal expression of cardiac IL-6 in SS mice, which was further elevated by increase in extracellular heme. Elevated IL-6 is associated with higher risk of left ventricular dysfunction and progression to heart failure in humans (38), and hypertrophy in rodents (39). Furthermore, in SCD patients LV dysfunction was an independent risk factor for death (40, 41), while diastolic dysfunction and myocardial fibrosis were reported in sickle cell mouse model (42). Therefore, we hypothesized that heme might induce cardiac hypertrophy genes in sickle cell mice. We evaluated expression of atrial natriuretic factor (Nppa) and β-Myosin heavy chain 7 (Myh7), known to be associated with cardiac hypertrophy (43). Baseline expression of both Nppa and myh7 were similar in the hearts of AA and SS mice (Supplementary Figure 2). Heme treatment resulted in a 14.8-fold increase in Nppa transcripts and 8.1-fold increase in Myh7 transcripts in the heart of SS mice 3 h after injection of heme (Figures 4A–C), but not AA control mice (Figure 4C). The heart in SCD appears to be more sensitive to heme induction of these two cardiac hypertrophy genes.

## Discussion

Intravascular hemolysis is an important modifier of outcome and pathogenesis of SCD (2). The plasma cell-free hemoglobin and heme are elevated at steady state in SCD and are associated with disease severity and end organ damage (2, 44). Cardiac-related complications represent a leading cause of death in SCD (26). In SCD patients, there is also a dysregulated expression of IL-6 and other inflammatory cytokines linked to vaso-occlusive crisis and other complications (18, 20). In this study, we report for the first time elevated basal cardiac IL-6 mRNA and protein levels in SS mice compared to AA controls. We also showed that experimental increase in circulating heme further elevates cardiac and plasma IL-6 expression in control mice and even more so in SS mice. Our result is consistent with earlier studies that reported elevated serum IL-6 in SCD patients (16, 18), but those studies did not investigate the heart. It is possible that cardiomyocytes and non-cardiomyocytes including fibroblasts and macrophages in the heart as well as cells in other organs may be contributing to the elevated plasma IL-6 after heme injection.

Hemopexin, the endogenous scavenger of free heme, is depleted from the serum of both human (45, 46) and mice (34, 47) with SCD, making them more susceptible to acute increases in heme concentration. This promotes the elevated circulating heme levels reported in human (35) and mice with SCD (34). Once the plasma heme scavenging system is saturated, circulating heme can generate reactive oxygen species, resulting in tissue injury. Our results indicate that heme also upregulates inflammatory cytokine IL-6. Our finding of heme induction of cardiac IL-6 complements a recent report of higher cardiac IL-6 transcripts in hemopexin deficient mice compared to wildtype control (48). The protective effect of hemopexin in heme injection experiments in sickle cell and hemopexin deficient mice has previously been published by our group (34, 49) and other authors (47, 50).

Our findings show that Nrf2 is dispensable for heme induction of IL-6 expression. Further investigation of this pathway was beyond the scope of this current investigation, with several possible mechanisms that might be involved in this heme response. Multiple regulatory elements in the promoter region of the IL-6 gene may contribute to its regulation in a cell type-specific manner (51). Nrf2 might act as a transcriptional repressor or it might regulate another transcriptional inhibitor of IL-6 expression (52). Additionally, reduced inducibility in Nrf2^−/−^ mice of cardiac Hmox1, the principal enzyme in heme catabolism (32), could result in slower degradation of heme leading to prolonged heme-induced activation of IL-6 through an Nrf2-independent pathway. Chronic long-term signaling of IL-6 induces inflammation and promotes cardiac hypertrophy in other models (53). Both features are risk factors for morbidity and mortality in SCD (29, 40, 54), although their relationship to each other has not been investigated in SCD.

Our pilot analysis of other cardiac mRNAs shows a concurrent induction of transcripts of cardiac hypertrophy genes Nppa and MyH7 by heme in the heart of SS mice. Additional inflammatory cytokines such as PlGF contributes to cardiac hypertrophy through IL-6 signaling (55) and it is a predictor of increased left ventricular mass in non-hemolytic diseases such as chronic kidney disease (56). We recently published evidence of elevated basal cardiac expression of PlGF in SS mice with further inducibility by heme (32). These results support a hypothetical model that chronic hemolysis induces expression of both PlGF and IL-6, and this elevation of inflammatory cytokines might contribute to the development of LVH in SCD through a yet to be experimentally identified mechanism. The association of hemolysis, IL-6 induction and organ damage in SCD is supported by previous published research. A recent report showed an association between a polymorphism in the IL-6 gene and development of leg ulcer in SCD patients (57), while previous publications showed hemolysis as a risk factor for leg ulcer in these patients (2, 58). Taken together, these suggest that increased hemolysis and inflammatory cytokines including IL-6 may play an important role in organ injury and pathophysiology of SCD.

Our study has several limitations. We did not evaluate the hemopexin levels in plasma or the heart and we did not test whether hemopexin would be protective against heme induction of IL-6. We did not determine a specific cell type in the heart in which heme activates IL-6 expression, and this is a future goal. The mechanism of heme-induced IL-6 expression remains to be determined, although our present evidence unequivocally demonstrates that Nrf2 is not required. An alternative mechanism might involve the activation of the Toll-like receptor 4 (TLR4) induction of MyD88, activator protein-1 (AP-1) and nuclear factor–κB (NF-κB) pathways (59–61). The specific mechanisms by which hemolysis-induced IL-6 contributes to the development of cardiac hypertrophy in SCD warrants future investigation. Despite these limitations, the findings of this study are novel and set the stage for detailed mechanistic studies of heme induction of cardiac IL-6 in SCD. This may lead to the development of novel therapeutic targets for ameliorating or preventing heme-induced IL-6 and cardiac dysfunction in SCD.

In conclusion, we show for the first time direct induction of IL-6 by heme in the plasma and heart of SS mice, in a mechanism that does not require Nrf2. We also show for the first time that heme induces cardiac expression of genes associated with cardiac hypertrophy, a clinically significant complication found in SCD patients with especially severe chronic hemolysis. These new observations provide the basis for a previously unknown heme/IL-6 axis in the development of cardiac disease in patients with SCD. This new model provides potential therapeutic targets for intervention in the heme response and IL-6 pathways to prevent cardiac disease in SCD that merit additional investigation.

## Data Availability Statement

The datasets generated for this study are available on request to the corresponding author.

## Ethics Statement

The animal study was reviewed and approved by University of Pittsburgh Institutional Animal Care and Use Committee (IACUC).

## Author Contributions

OG and GK designed the research project, analyzed, interpreted data, and drafted the manuscript. OG performed the experiments. MK and SG assisted with the experiments. FV and SO-A supervised experiments, and interpreted data. All authors critically reviewed and approved the final version.

## Conflict of Interest

The authors declare that the research was conducted in the absence of any commercial or financial relationships that could be construed as a potential conflict of interest.

## Supplementary Material

Supplementary Figure 1 GUSB expression in vehicle and haem-injected mice. Endogenous GUSB expression in vehicle and hemin-treated AA mice (*n* = 3–6). For C_t_, lowest value = highest expression and highest value = lowest expression. All values are mean ± SEM. Exp, Expression. There were no significant differences between vehicle control and hemin treated values.

Supplementary Figure 2 Similarity in baseline transcripts of markers of cardiac hypertrophy in AA and SS mice. Baseline expression of Nppa and Myh7 in the heart of AA and SS mice. For DeltaC_t_, lowest value = highest expression and highest value = lowest expression. Target gene transcripts were normalized to Gusb for all mRNA expression level. All values are mean ± SEM. Exp, Expression.
